# P2x7 deficiency suppresses development of experimental autoimmune encephalomyelitis

**DOI:** 10.1186/1742-2094-5-33

**Published:** 2008-08-08

**Authors:** Anthony J Sharp, Paul E Polak, Vittoria Simonini, Shao X Lin, Jill C Richardson, Ernesto R Bongarzone, Douglas L Feinstein

**Affiliations:** 1 Department of Anesthesiology, University of Illinois, Chicago, IL 60612, USA; 2 Jesse Brown Veteran's Affairs Research Division, Chicago, IL 60612, USA; 3 GlaxoSmithKline Pharmaceuticals, Harlow, UK; 4 Department of Cell Biology and Anatomy, University of Illinois, Chicago, IL 60612, USA

## Abstract

**Background:**

The purinergic receptor P2x7 is expressed on myeloid cells as well as on CNS glial cells, and P2x7 activation has been shown to increase both glial and T-cell activation. These properties suggest a role in the development of autoimmune disease including multiple sclerosis.

**Methods:**

The animal model of MS, experimental autoimmune encephalomyelitis (EAE) using myelin oligodendrocyte glycoprotein (MOG) peptide residues 35–55 was induced in wildtype C57BL6 mice and in P2x7 deficient mice ('P2x7 mice') that were backcrossed to C57BL6 mice. Disease progression was monitored by appearance of clinical signs, immunocytochemical staining to assess brain inflammation and neuronal damage, and by measurement of Tcell cytokine production.

**Results:**

The incidence of EAE disease in P2x7 mice was reduced 4-fold compared to the wildtype mice; however the P2x7 mice that became ill had similar days of onset and clinical scores as the wildtype mice. Splenic T-cells isolated from P2x7 null mice produced greater IFNγ and IL-17 (from 3 to 12 fold greater levels) than wildtype cells, however cytokine production from P2x7 derived cells was not increased by a selective P2x7 agonist as was cytokine production from wildtype cells. Although infiltrating cells were detected in brains of both the P2x7 and wildtype mice, astroglial activation and axonal damage was reduced versus wildtype mice, and the distribution of astroglial activation was markedly distinct in the two strains. In contrast, microglial activation was similar in the two strains.

**Conclusion:**

P2x7 deficiency resulted in compensatory changes leading to increased T-cell cytokine production, and activated T-cells were detected in the brains of P2x7 null mice with no clinical signs. However, the greatly reduced incidence of disease suggests that an initiating event is absent in these mice, and points to a role for astroglial P2x7 in development of EAE disease.

## Background

Multiple sclerosis (MS) is an autoimmune demyelinating disease in which activated T-lymphocytes infiltrate the CNS, secrete pro-inflammatory substances which activate parenchymal glial cells, and ultimately lead to oligodendrocyte and neuronal damage. During this process, cell damage or death can lead to increased levels of extracellular ATP, which can act upon purinoceptors on neurons and glia. The P2X7 receptor is a ligand-gated ion channel predominantly expressed on immune cells of the periphery (mainly macrophages) and CNS (microglia), but has also been detected in astrocytes [1-3], oligodendrocytes [4,5] and neurons [6,7]. P2X7 is activated by high concentrations (in the 100 μM range) of ATP and prolonged agonist activation leads to the formation of a large cytolytic pore in the cell membrane. Activation of P2X7 causes depolarization, leading to an increase in K+ efflux, and eventual activation of the inflammasome (a large multiprotein complex implicated in caspase-1 and caspase-5 activation [8]. As a consequence, caspase-1 is activated leading to a rapid maturation and release of the potent pro-inflammatory cytokine IL-1β, a key initiator of the inflammatory cascade [9-11]. Activation of P2x7 in brain can also increase glial calcium and sodium influx [12], increase reactive oxygen and nitrogen species [13,1,14]; modulate glial efflux of glutamate and ATP [2,3,15] and has been associated with neuronal damage [16,17].

P2x7 activation plays a role in the regulation of lymphocyte activation. Activation of P2x7 by ligands including ATP, by transfer of ribosyl groups from extracellular NAD+ to P2x7 [18,19], or by synthetic ligands including 2',3'-(benzoyl-4-benzoyl)-ATP (BzATP) induces pore formation, Ca^2+ ^influx, annexin V staining, and cell death in T-cells [20], natural killer cells (NKTs, [21], and regulatory T-cells (Tregs, [22]). Interestingly, in some cases it was the unprimed, naïve cells that were more sensitive to P2x7 dependent cell death [20,21], while the activated cells were resistant and instead were further activated by P2x7. Thus, P2x7 activation can result in either reduced or exacerbated lymphocyte responses [23].

The above findings suggests that increases in extracellular levels of ATP or NAD+ which could occur during MS could contribute to inflammatory damage by activating P2x7, and studies using P2x7 antagonists or deficient mice support this idea. In macrophages, it was shown that while responses to LPS (an increase in cyclooxygenase 2 expression and pro-IL1β production) was similar in wildtype (WT) and P2x7 null mice, the increased response to ATP (increased externalization and processing of pre-IL1β to mature IL1β; and subsequent increase in IL-6 expression) was absent in the null mice [24]. In vivo, prolonged activation of P2x7 (accomplished by infusion of the potent and selective ligand BzATP) resulted in tissue damage, gliosis, and axonal damage reminiscent of EAE; while treatment of EAE mice with P2x7 antagonists ameliorated clinical symptoms and reduced demyelination [4]. However, characterization of EAE disease in P2x7 null mice [25] unexpectedly showed more severe disease in the null mice versus wildtype controls (C57Bl6 mice), associated with a greater number of lesions in brain, increased lymphocyte proliferation and reduced lymphocyte cell death, and reduced splenic cell production of pro-inflammatory cytokines including IL-6 and IFNγ. In a related study [26], the authors reported that axonal damage in P2x7 null mice was increased compared to that in the WT mice. These results led to the conclusion that P2x7 is important for regulating lymphocyte numbers and subtypes, and therefore critical to the development of EAE disease.

In the current study, we have re-evaluated the consequences of P2x7 deficiency on the progression of MOG peptide induced EAE disease using a distinct strain of P2x7 null mice [27-29]. In contrast to the mice used for previous EAE studies, in which deficiency was achieved by homologous recombination of the neomycin gene into exon 5 [24], in the current studies P2x7 deficiency was achieved by neomycin insertion into exon 1 [27]. In these mice, the conversion of pro-IL-1β to mature, released IL-1β is absent from activated macrophages, demonstrating the absence of P2x7 dependent IL-1β production. However whether other cell types express any P2x7 dependent responses was not determined. In the current study, we found that the overall incidence of EAE disease was significantly reduced in P2x7 null mice compared to controls. However, cytokine production from splenic T-cells isolated from immunized P2x7 null mice was greater than that observed from WT cells, suggesting a compensatory response to P2x7 deficiency. Within the CNS, astroglial activation was present but reduced in the P2x7 null mice; and axonal damage less. These results are consistent with the idea that activation of P2x7 in parenchymal astrocytes cells is a main contributor to disease pathogenesis, and therefore treatment with CNS-penetrant P2x7 antagonists should be considered as a therapeutic option in MS.

## Methods

### Mice

P2x7 deficient mice were described previously and were obtained from Glaxo-Smith-Kline (Harlow, Essex) [27]. These mice have been backcrossed onto a C57BL6 background. As controls, we used female C57BL/6 mice, aged 6–8 weeks from Charles River Breeding (Cambridge, MA). Mice were maintained in a controlled 12:12 hr light/dark environment and provided food ad libitum. All experiments were approved by the local animal care committee.

### Induction of EAE

EAE was actively induced in C57BL/6 and P2x7 null mice using synthetic myelin oligodendrocyte glycoprotein peptide 35–55 (MOG_35–55_) as described [30]. The MOG_35–55 _peptide (MEVGWYRSPFSRVVHLYRNGK) was obtained from Anaspec (San Jose, CA), and purified to >98% purity by reverse phase HPLC analysis. Mice were injected subcutaneously (two 100 μL injections into adjacent areas in one hind limb) with an emulsion of 300 μg MOG_35–55 _dissolved in 100 μL PBS, mixed with 100 μL CFA containing 500 μg of Mycobacterium tuberculosis (Difco, Detroit, MI). Immediately after MOG_35–55 _injection, the animals received an i.p. injection of pertussis toxin (PT, 200 ng in 200 μL PBS). Two days later the mice received a second PT injection, and 1 week later they received a booster injection of MOG_35–55_. Clinical signs were scored on a 5 point scale: grade 0, no clinical signs; 1, limp tail and/or impaired righting; 2; paresis of one hind limb; 3; paresis of two hind limbs; 4; moribund; 5, death. Scoring was done at the same time each day by a blinded investigator.

### T-cell isolation and cytokine measurements

Splenocytes were isolated from MOG_35–55 _immunized EAE mice at 4 days or 21 days after the booster MOG injection. After lysis of red blood cells, splenocytes were plated into 24 well plates at a density of 2 × 10^5^cells per well in 400 μl RPMI media, and incubated with immunogen (0 or 25 μg/ml MOG _35–55 _peptide). After 1 day, aliquots of the media were assayed for levels of IL-17 or IFNγ by ELISA following recommended procedures (Quantikine M, R&D Systems, Minneapolis, MN). Each sample was assayed at least in triplicate, calculation of ng/ml cytokine determined from standard curves, and the increase due to the presence of MOG peptide was determined. A semi-quantitative analysis of 64 inflammatory molecules produced by activated splenocytes was carried out using the mouse cytokine antibody array III (RayBiotech) according to the manufacturer's protocols.

### Analysis for infiltrating cells

Mouse brains were perfused with saline and post fixed in 4% paraformaldehyde overnight. Dehydration of hemispheres was through a series of 80%, 95% ethanol one hour each followed by 100% ethanol overnight. Two 100% xylene washes were done for 1 hour each and then 1 hour in 60°C Paraplast Plus (Tyco/Healthcare, Mansfield, MA). After a change of Paraplast Plus, tissue was placed in a 60° vacuum oven for 2 hours prior to placing in molds to cool and solidify. Tissue was cut 8 μm thick. Sagital sections (8 or 10 μM) were cut beginning from midline, and mounted. Sections were deparaffinized by drying overnight on superfrost plus slides (Fisher), heating at 56°C for 2 hr, and then washing through mixed xylenes, 100% ethanol, 95% ethanol, ddH_2_O, and then stained with hematoxylin and eosin. Four serial sections from each animal were analyzed for the number of infiltrating cells present in the region extending from the olfactory bulb to the dorsal third ventricle using Zeiss Axiovision 4.3 software. Black and white images of all stained sections were obtained using the same exposure time with a 10× objective. Specific fields containing small round hematoxylinophyllic nuclei were defined, and contrast adjusted so as to not count larger round cells presumably oligodendrocytes. The software automatically counts the number of independent objects (nuclei) within the field of interest; and data is presented as number of cells per mm^2^.

Immunohistochemisry was used to detect T-cells. For this, frozen sections were stained using an anti-CD8 antibody (Pharmingen, Mouse IgG, 1:500). Sections were incubated in 3% BSA in PBS plus antibody overnight at 4°C, washed, and then developed with a secondary antibody labeled with FITC.

### Immunohistochemistry

Sagittal mouse brain sections mounted on slides and deparaffinized as above, Antigen retrieval was accomplished by boiling for 10 minutes in 10 mM NaCitrate pH 6.0 and allowing slides to gradually cool to room temperature. Slides were then washed three times for 2 min. with ddH_2_O, then washed 5 min with PBS and blocked with 5% normal donkey serum in PBS at room temperature for 30 min. Primary antibodies were diluted in 1% normal donkey serum: mouse monoclonal antibody anti-SMI-32 1:200 (Sternberger Monoclonals Inc., Lutherville, MD); rabbit anti-MBP 1:300 (Zymed Laboratories, San Francisco, CA); rat anti-human GFAP monoclonal antibody B2.210 1:300 (a gift of V. Lee), and sections were incubated 4°C overnight. Sections were washed 3 times with PBS. Secondary antibodies (Jackson ImmunoResearch Inc., West Grove, PA) were donkey anti-rabbit RRX conjugated, donkey anti-rat FITC conjugated, or mouse on mouse kit conjugated with FITC (Vector Labs, Burlingame, CA). All secondary antibodies were incubated for 1 hour at 37°C and diluted 1:200 in PBS with 1% normal donkey serum. Slides were washed 3 times 5 minutes with PBS and post-fixed in 3.7% formaldehyde in PBS for 20 min. DAPI staining of nuclei, 400 ng/ml in PBS, was done after one wash with PBS and followed with 3 PBS washes. Vectashield mounting fluid (Vector Laboratories Inc., Burlingame, CA) was used. Images were obtained on a Zeiss Axioplan2 fluorescence microscope equipped with an Axiocam MRm digital camera and Axiovision 4.3 imaging software. For each analysis, at least 3 mice from each group were included, and 3–4 serial sections were analyzed from each mouse.

To detect activated microglia, free floating cryosections (8 μm) were immunostained using the F4-80 clone (Chemicon, rat IgG 1:200). Briefly, sections were rinsed in PBS and endogenous peroxidase quenched by incubation in methanol:hydrogen peroxide before blocking in 3% BSA in PBS. After overnight incubation with F4-80 at 4°C, sections were processed for biotin-streptoavidin-peroxidase staining as recommended by the manufacturer (Vector Labs). Sections were developed using diaminobenzidine and hydrogen peroxide.

### Data analysis

The data from 3 separate EAE studies of P2x7 null mice and 2 separate studies done in WT mice was combined and analyzed. Data is presented as the mean ± sem, and differences between group averages were determined by unpaired, nonparametric T-test with P < 0.05 considered significant. Data in figures 2 and 3 are presented as mean ± sd. The effects of BzATP treatment on cytokine production were analyzed by 1-way ANOVA and Bonferroni's multiple comparison post hoc test.

**Figure 1:**
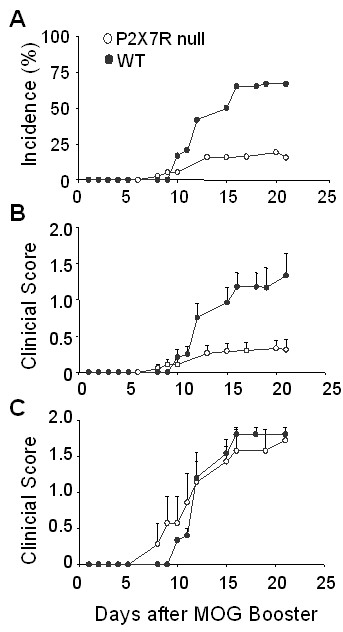
**Effect of P2x7 deficiency on the development of clinical signs of EAE**. P2x7 null and wildtype C57BL6 mice were immunized to develop EAE using MOG_35–55 _peptide, and clinical scores monitored approximately daily. Day 0 is defined as the day of the MOG peptide booster immunization. The data from 3 separate studies using P2x7 null mice (n = 38 total) and 2 separate studies using WT mice (n = 24 total) are combined. Data in (**A**) shows average incidence of disease (defined by at least 2 consecutive days with a score of 1.0 or greater). Data in (**B**) shows average clinical scores (mean ± se) for all mice. Data in (**C**) show average clinical scores (mean ± se) for only those mice that became ill (7/38 in the P2x7 null group; and 15/24 in the WT group).

**Figure 2:**
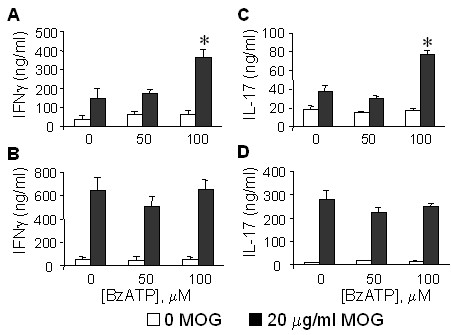
**Effect of P2x7 deficiency on splenic T-cell cytokine production**. P2x7 and wildtype C57BL6 mice were immunized to develop EAE as above, and at 4 days after the booster immunization, splenic T-cells isolated and cultured in the presence (filled bars) or absence (open bars) of 20 μg/ml MOG peptide, and either 0, 50, or 100 μM BzATP. After 24 hr, levels of IFNγ and IL-17 in the culture media were determined by specific ELISA. Cytokine production is shown for WT (**A**, **C**) and P2x7 null (**B, D**) cells. Values are the mean ± sd of n = 3 independent samples. *, P < .001 versus no BzATP.

**Figure 3:**
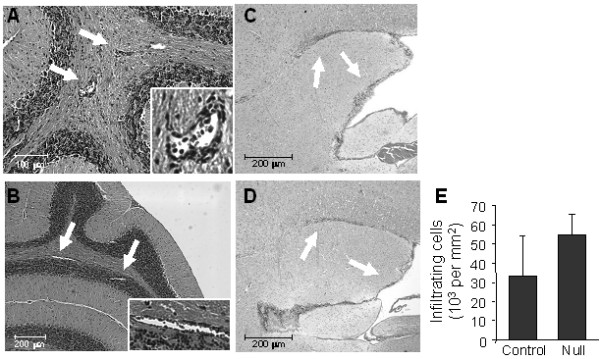
**Histological staining for infiltrating cells in P2x7 and WT mice**. Serial sections were prepared from EAE immunized P2x7 null and WT mice at day 21 after immunization. At this time, the 3 WT mice had clinical scores of 2.0; and the 3 P2x7 null mice had no clinical signs. Sections were stained with H&E to detect the presence of infiltrating cells. Representative sections from the cerebellum show the presence of perivascular infiltrating cells (arrows) in the cerebellar white matter of both P2x7 (**A**) and WT (**B**) brains. Sections taken from near the IVth ventricle show the presence of infiltrating cells (arrows) beneath the corpus callosum and lining the ventricle in both P2x7 (**C**) and WT (**D**) brains. (**E**) Quantitation of the average total number of infiltrating cells (mean ± sd) measured in 4 serial sections for P2x7 and WT mice (n = 3 mice each).

## Results

### P2x7 deficiency suppresses clinical symptoms in EAE

The clinical course of EAE disease was monitored in P2x7 null and wildtype (WT) C57BL/6 mice (Figure 1). In data obtained from 3 separate studies (Table 1), the overall incidence of disease (Figure 1A) was significantly decreased (to 7/38 or 18%) in P2x7 null mice compared to controls (15/24 or 63%); although the average day of onset for those mice that became ill was similar to the WT mice. Comparison of the clinical scores for all mice (ill and non-ill, Figure 1B) shows that the average scores were significantly lower in the P2x7 null group compared to WT group; however the development of clinical disease and maximum scores of the mice that became ill in each group were similar (Figure 1C). These data suggest that absence of P2x7 significantly reduces the incidence of EAE, but those mice that become ill have a similar disease course do the WT mice.

**Table 1:** Clinical symptoms in P2x7 null and C57Bl6 wildtype mice

**Study**	**Incidence**	**Mean Onset**	**Maximal** ** Score All**	**Maximal** ** Score ill**
*P2x7 null*				
Expt 1	4/18 (22%)	10.8 ± 1.3	0.4 ± 0.2	2.0 ± 0.0
Expt 2	0/8 (0%)	nd	0	0
Expt 3	3/12 (25%)	14.3 ± 2.3	0.4 ± 0.3	1.3 ± 0.2
Total	7/38 (18%)	12.3 ± 1.2	0.3 ± 0.1	1.7 ± 0.2
*C57BL/6*				
Expt 1	7/12 (64%)	12.7 ± 0.6	1.2 ± 0.3	1.9 ± 0.1
Expt 2	8/12 (67%)	12.7 ± 1.0	1.4 ± 0.3	2.1 ± 0.1
Total	15/24 (63%)	12.7 ± 0.6	1.2 ± 0.7	1.9 ± 0.1

### Effects of P2x7 deficiency on T-cell activation

Antigen restimulation of cytokine production was measured in splenic T-cells isolated from EAE immunized mice 4 days after the booster immunization (Figure 2). We noted that the spleens from P2x7 null mice were larger than those from WT mice, and the total yield of splenocytes about twice as great (not shown), consistent with the report that P2x7 null splenocytes proliferated more than the WT cells [25]. T-cells from the WT mice (Figure 2A) produced basal levels of IFNγ (39.9 ng/ml) which were increased about 4-fold by MOG peptide (150 ng/ml). Cells from P2x7 null mice (Figure 2B) produced similar basal levels of IFNγ (54 ng/ml) which were increased to a greater extent (about 12-fold) by MOG than in the WT cells. WT cells also produced basal levels of IL-17 (18 ng/ml, Figure 2C) which were increased about 2-fold by MOG; whereas P2x7 null cells (Figure 2D) basally produced about 11 ng/ml IL-17 which was increased almost 25-fold by MOG. These results demonstrate that P2x7 null T-cells are efficiently primed and respond to antigen to the same or greater extent than do WT-derived cells.

We compared the effects of the selective P2x7 agonist BzATP on splenocyte responses to ex vivo restimulation with MOG peptide. In WT cells, co-incubation with 100 μM BzATP significantly increased both IFNγ and IL-17 production about 2-fold (Figure 2A, C). In contrast, BzATP did not increase cytokine production from P2x7 null cells (Figure 2B, D). This suggests that despite a compensatory effect leading to increased T-cell activation, the lack of a functional P2x7 limits further potentiation by selective ligands.

The increased response of P2x7 null splenocytes was not limited to IL-17 and IFNγ, since a membrane-based ELISA assay showed that P2x7 null derived splenocytes produced increased levels (50% or greater) of several other inflammatory factors including IL-12p40 subunit, MCP1, CXCL16, IL-3 and it's receptor IL-3Rb, L-selectin, and eotaxin (data not shown).

### Effects of P2x7 deficiency on brain histopathology

**Figure 4:**
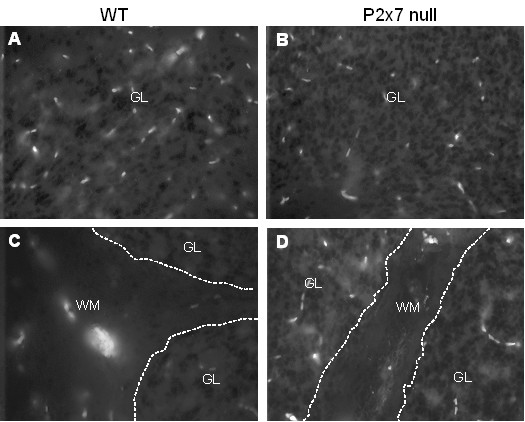
**CD8 T-cells are expressed in WT and P2x7 cerebellum**. Serial sections as prepared in Figure 3 were stained with antibody to CD8 to detect the presence of T-cells. Representative images from one mouse in each group show the presence of CD8 cells in both the granule layer ('GL', Panels **A **and **B**) and white matter ('WM', Panels **C **and **D**) of WT and P2x7 animals.

Histological analysis revealed the presence of infiltrating cells throughout the brain of P2x7 null mice that did not develop any clinical signs, including cells located in small perivascular lesions in the cerebellum (Figure 3A, B) and beneath the corpus callosum and lining the lateral ventricles (Figure 3C, D). These cells represent infiltrating lymphocytes as previously shown [30]. A quantitative assessment of the latter showed a similar, if not greater number of infiltrating cells in P2x7 brains as in the WT brains (Figure 3E). Immunocytochemical staining of cerebellum revealed the presence of CD8 positive Tcells in the white matter and granule layers of both WT (Figure 4A, C) and P2x7 (Figure 4B, D) mice.

**Figure 5:**
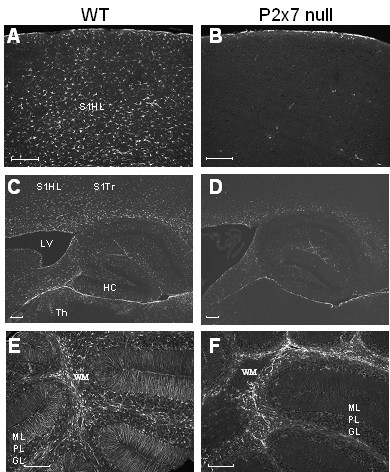
**GFAP expression is reduced in P2x7 brain**. Serial sections were prepared from EAE immunized P2x7 null and WT mice at day 21 after immunization. At this time, the 3 WT mice all had clinical signs of 2.0; and the 3 P2x7 null mice had no clinical signs. Sections were stained with antibody to GFAP to detect activated astrocytes. Representative images show robust GFAP staining in WT frontal cortex (**A**) and thalamus (**C**), but not in corresponding areas of P2x7 null cortex (**B**) or thalamus (**D**). Significant staining for GFAP was found throughout the cerebellar grey and white matter in the WT mice (**E**) but was restricted to white matter (W) in P2x7 mice (**F**). S1HL, somatosensory hindlimb region; S1Trl, somatosensory trunk region; LV, lateral ventricle; HC, hippocampus; Th, laterodorsal thalamic nuclei; WM, white matter; ML, molecular layer; PL, Purkinje cell layer; GL, granule cell layer. Size bars are 200 μm.

Astrocyte activation, assessed by staining for GFAP was found throughout the brains of WT mice at the peak of clinical disease (Figure 5). In contrast, while some GFAP staining was detected in the brains of P2x7 null mice, it was reduced compared to WT mice in many regions including the somatosensory region of cortex projecting to hindlimb and trunk areas (Figure 5A, B), corpus callosum, and underlying thalamic area (Figure 5C, D). In the cerebellum of WT EAE mice, GFAP staining was detected in the white matter as well as in Bergman radial glial fibers (Figure 5E), while in P2x7 null mice robust GFAP staining was only observed in cerebellar white matter (Figure 5F). These data suggest that parenchymal astrocyte activation is reduced in the knockout mice compared to WT mice.

**Figure 6:**
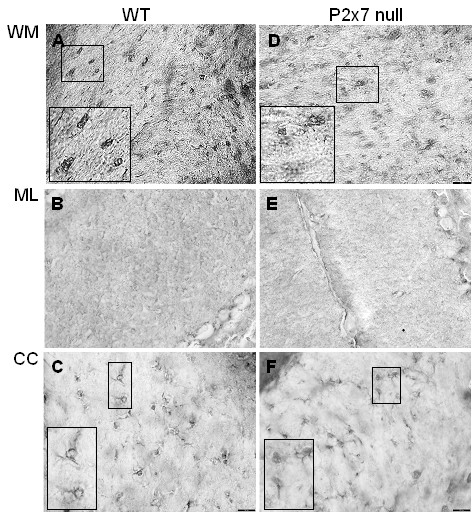
**Microglial activation is similar in P2x7 and WT brain**. Serial sections from WT (**A**, **B**, **C**) and P2x7 null (**E**, **F**, **G**) mice as described in Figure 5 were stained with monoclonal antibody F4/80 to detect activated microglia and macrophages. Representative images from one mouse in each group show the presence of cells with characteristic morphology of microglia in cerebellar white matter ('WM', panels **A**, **D**) and corpus callosum ('CC', panels **C**, **F**); and diffuse staining in cerebellar granule layer ('GL', panels **B**, **E**).

In contrast to astrocyte activation, immunostaining using monoclonal antibody F4/80, a marker of activated microglia and macrophages, revealed similar levels and patterns of microglia activation in WT and P2x7 null mice (Figure 6). In the cerebellum, strongly stained cells with typical microglial morphology were observed in white matter of WT and P2x7 null mice (Figure 6A, D), and diffuse staining in the molecular layer (Figure 6B, E). Robust microglial activation was also observed in the corpus callosum of both strains (Figure 6C, F). In contrast to GFAP, we did not observe strong F4/80 staining any any cortical areas (not shown). These results suggest that microglial activation is similar in P2x7 null and WT mice at the time point examined.

**Figure 7:**
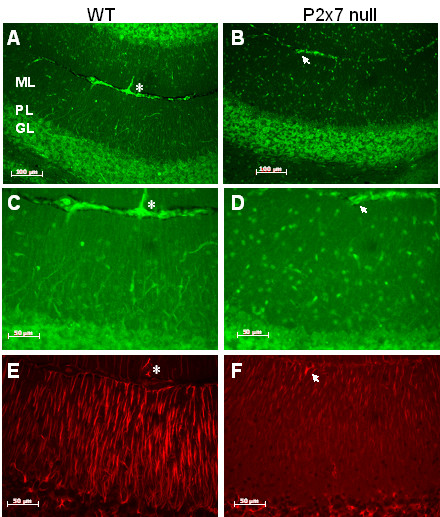
**Axonal damage is reduced in P2x7 brain**. Serial sections were prepared from EAE immunized P2x7 null and WT mice at day 21 after immunization. At this time, the 3 WT mice all had clinical signs of 2.0; and the 3 P2x7 null mice had no clinical signs. Sections were stained with antibody SMI32 (for non phosphorylated neurofilament proteins, **A-D**) or to GFAP (**E**, **F**). Representative images show significant SMI32 staining in an area of the cerebellum from WT (**A**, **C**) mice, but little staining in a comparable area from P2x7 null (**B**, **D**) mice. Size bars are 100 μM (**A**, **B**) and 50 μm (**C-F**). GFAP staining (**E**, **F**) illustrates the differences in localization for GFAP and SMI32 staining. Asterisks and arrows are placed to indicate same location in images. ML, molecular layer; PL, Purkinje cell layer; GL, granule cell layer.

Immunostaining for the presence of non-phosphorylated neurofilament protein revealed axonal damage in the cerebellar molecular layer in WT mice (Figure 7A), emanating outwards from the strongly stained Purkinje cell layer (Figure 7C), and having a distinct localization from that of the increased GFAP staining (Figure 7E). In contrast, axonal damage in the molecular layer was minimal in the the P2x7 null mice (Figure 7B, D), as was staining for activated astrocytes (Figure 7F).

## Discussion

Our data demonstrate that P2x7 deficiency significantly reduces the incidence of EAE disease compared to control C57BL6 mice; however the mice which developed disease (7 of 38 total) showed a similar day of onset and reached similar disease severity as the ones that became ill in the control group. This suggests that the presence of P2x7 is necessary for efficient initiation of disease, but that disease can occur, albeit at a reduced level, in the absence of P2x7. The nature of the initiating event (or events) is not known, but our findings suggest that it does not involve T-cell activation or their ability to infiltrate into the CNS; but instead reflects a difference in inflammatory responses of parenchymal astrocytes.

Despite significantly reduced disease incidence, we found that cytokine production from splenocytes isolated from P2x7 null mice that did not show any clinical signs was as high as or greater than that measured in splenocytes obtained from C57BL/6 mice that exhibited clinical signs. While basal production of IFNγ and IL-17 was similar, restimulation with MOG peptide led to significantly greater cytokine production in the P2x7 null cells compared to controls. This finding suggests that P2x7 deficiency can lead to increased T-cell activation, although whether it is an effect on the Tcells or other cells present (e.g. dendritic cells) is not known. A recent study reported that while activation of P2x7 increased cytokine production from activated Natural Killer T-cells (NKTs), it also increased apoptosis of naïve NKT cells [21]. Therefore, increased T-cell activation could be due to reduced death of regulatory NKT cells. Our data also demonstrates that, in contrast to WT cells, the P2x7 null cells do not increase cytokine production in response to BzATP. It is therefore possible that within the context of an inflammatory milieu as exists in EAE, increased extracellular levels of ATP (or of other agonists which can activate P2x7) can promote cytokine production from WT cells but not from P2x7 deficient cells.

Despite the increased ability of P2x7 null T-cells to produce cytokines, and the presence of lymphocytes in the brains of P2x7 null mice, the majority of the mice showed no clinical signs, suggesting that parenchymal production of inflammatory factors is attenuated. We used GFAP staining as an index of astrocyte activation, and observed that while there is extensive astrocyte activation though out the brains of C57BL6 mice that showed clinical signs, there was limited GFAP staining in the P2x7 null mice. In particular, we noticed that in the cerebellum of the control mice, GFAP staining was present in the white matter area as well as in processes of radial glial cells extending through the molecular layer. In contrast, GFAP staining in the P2x7 null mice was mainly observed in the cerebellar white matter. These observations indicate that astroglial activation does occur in the P2x7 null mice but in a restricted manner compared to controls.

In contrast to a clear differential degree of astrocyte activation, staining using monoclonal antibody F4/80 did not reveal significant differences in microglial activation between WT and the P2x7 null mice, suggesting an important role for astroglial P2x7 during EAE disease progression. There are several ways by which lack of P2x7 from astrocytes, could provide benefit during EAE that may not be shared with microglial cells. Astrocytes, but not microglia, have been shown to release purines following P2x7 activation [31], which could contribute to ongoing inflammation. In astrocytes, P2x7 activation induces ERK1/2 phosphorylation [32] and activation of the PI3/Akt signaling system [33], both which can contribute to increase inflammatory responses; but this has not been demonstrated to occur in microglial cells. An important function of astrocytes is to remove excessive glutamate from the extracellular environment, which could otherwise contribute to neuronal damage. A recent study [34] demonstrates that activation of P2x7 receptors on astrocytes reduces glutamate uptake as well as glutamine synthetase. Similarly, it has been shown that P2x7 activation can induce glutamate release from astrocytes [35]. In a recent study [36], it was shown that astrocytes express a greater repertoire and higher levels of excitatory amino acid transporters than do microglial cells; and astrocytes but not microglia prevented excito-neurotoxicity due to glutamate. Astrocytes, but not microglia, have been shown to express the glutamate transporter GLT-1, as well as high levels of glutamate metabolizing enzymes in post mortem MS brain samples [37]. Together these findings suggest that astroglial regulation of extracellular glutamate levels may be of greater importance during neurodegenerative diseases, and therefore perturbation of astrocyte glutamate uptake by activation of P2x7 could have more drastic consequences.

The question arises as to why in a previous study P2x7 deficiency exacerbated EAE disease [25]. As noted above, the mice used in that study were generated with a strategy in which the recombination event targeted P2x7 exon V, while in the current study the neomycin cassette was inserted into exon I. This raises the possibility that an alternate form of P2x7 is expressed thus leading to a distinct phenotype. It has been reported that a variant of P2x7 is expressed in certain cell types [38,6]. If so, an alternate form of P2x7 in T-cells could contribute to increased cytokine production. This is also consistent with the fact that the P2x7 gene that was targeted for knockout in the founding 129 mouse line was the 415P allele, which is known to be more active than the 415L present in the C57Bl6 background [39]. While identification of possible alternative P2x7 transcripts remains to be determined, these studies confirm an important role for this receptor during the course of EAE, and by inference, MS disease.

## Abbreviations

BzATP: 2',3'-(benzoyl-4-benzoyl)-ATP; CFA: Complete Freund's adjuvant; DAPI: 4',6-diamidino-2-phenylindole; EAE: Experimental autoimmune encephalomyelitis; FITC: Fluorescein isothiocyanate; GFAP: Glial fibrillary acidic protein; IFN: Interferon; IL: Interleukin; MBP: Myelin basic protein; MOG: Myelin oligodendrocyte glycolprotein; PT: Pertussis toxin; RRX: Rhodamine red-X; RPMI: Roswell Park Memorial Institute.

## Competing interests

Dr Feinstein has received funding from GlaxoSmithKline which supported these studies. Dr Richardson is an employee of GlaxoSmithKline.

## Authors' contributions

DLF organized and designed studies, analyzed data, and drafter the manuscript. AJS carried out EAE immunizations and Tcell studies. PEP assisted with immunostaining. SXL bred and genotyped mice. MVS assisted with immunostaining and PCR analyses. JCR assisted in experimental design, data analysis, and manuscript preparation. EB assisted with immunostaining. All authors read and approved the final manuscript.
